# Toxicity and Biokinetics of Colloidal Gold Nanoparticles

**DOI:** 10.3390/nano5020835

**Published:** 2015-05-21

**Authors:** Mi-Rae Jo, Song-Hwa Bae, Mi-Ran Go, Hyun-Jin Kim, Yun-Gu Hwang, Soo-Jin Choi

**Affiliations:** 1 Department of Food Science and Technology, Seoul Women’s University, 621 Hwarang-ro, Nowon-gu, Seoul 139-774, Korea; E-Mails: mirae8651@naver.com (M.-R.J.); songhwa29@naver.com (S.-H.B.); miran8190@naver.com (M.-R.G.); kimhj043@naver.com (H.-J.K.); 2 SMNANOBIO Co., Ltd., 160 Techno 2-ro, Yuseong-gu, Daejeon 305-500, Korea; E-Mail: ykhwang80@gmail.com

**Keywords:** gold nanoparticle, cytotoxicity, oral absorption, tissue distribution, oral toxicity

## Abstract

Gold nanoparticles (Au-NPs) have promising potential for diverse biological application, but it has not been completely determined whether Au-NP has potential toxicity *in vitro* and *in vivo*. In the present study, toxicity of Au-NP was evaluated in human intestinal cells as well as in rats after 14-day repeated oral administration. Biokinetic study was also performed to assess oral absorption and tissue distribution. The results demonstrated that Au-NP did not cause cytotoxic effects on cells after 24 h exposure in terms of inhibition of cell proliferation, membrane damage, and oxidative stress. However, when a small number of cells were exposed to Au-NP for seven days, colony forming ability remarkably decreased by Au-NP treatment, suggesting its potential toxicity after long-term exposure at high concentration. Biokinetic study revealed that Au-NP slowly entered the blood stream and slightly accumulated only in kidney after oral administration to rats. Whereas, orally administered Au ions were rapidly absorbed, and then distributed in kidney, liver, lung, and spleen at high levels, suggesting that the biological fate of Au-NP is primarily in nanoparticulate form, not in ionic Au. Fourteen-day repeated oral toxicity evaluation showed that Au-NP did not cause severe toxicity in rats based on histopathological, hematological, and serum biochemical analysis.

## 1. Introduction

Colloidal gold nanoparticles (Au-NPs) have been extensively developed for various biomedical applications, such as biosensors, cancer cell imaging, photothermal therapy, drug delivery, and radiotherapy due to their easy surface modification as well as electronic, and optical properties [1,2,3]. Moreover, Au-NPs are currently used in cancer therapy and in the treatment of rheumatoid arthritis [4,5]. Traditionally, gold (Au) has been considered inert and biocompatible. However, nanoparticles possess unique properties different from bulk-sized materials, including large surface area to volume ratios, high reactivity, and strong interaction with biological matrices, related to their small size. Hence, the question as to whether Au-NPs have potential toxicological effects on human health remains to be answered.

Studies on the toxicity of Au-NPs have been reported *in vitro* and *in vivo* [6,7]. However, conflicting results have been demonstrated, depending on experimental conditions, such as cell lines, particle size, surface chemistry, concentration, and animal models used [8,9]. Chueh *et al.* [10] reported that cytotoxicity of Au-NP differed from mammalian cell lines, showing apoptosis in Vero cells originated from African green monkey kidney, genotoxicity in human normal lung fibroblast MRC-5 cells, and autophagy in mouse embryonic fibroblast NIH3T3 cells. Kodiha *et al.* [11] demonstrated that cetyltrimethyl ammonium bromide (CTAB)-coated Au nanosphere (15.6 ± 1.6 nm) and Au nanoflowers (40–120 nm), but not big PEGylated Au nanosphere (86 nm), caused nuclear damage in breast cancer MCF7 cells. On the other hand, small number of toxicity research on Au-NPs was performed *in vivo* in animal models. Kim *et al.* [12] examined the effects of surface charge and particle size of Au-NP on an embryonic zebrafish model. Toxicity of Au-NP (13.5 nm) was also demonstrated in mice by oral, intravenous, and intraperitoneal injection, respectively, showing that tail vein injection of Au-NP had less toxicological effects than oral or intraperitoneal injection [13]. Indeed, most *in vivo* toxicity studies of Au-NP have been investigated by intraperitoneal or intravenous injection, whereas its toxicity by oral exposure has not been extensively explored. However, the application potential of Au-NPs in oral delivery carriers or therapeutic agents have been focused on and need further verification on their oral toxicity [14,15]. Moreover, biokinetic behaviors of Au-NPs, including oral absorption efficiency and tissue distribution pattern, have not been well determined, which can provide fundamental information on their potential toxicity and biomedical application at safe levels.

The aim of this study was, therefore, to evaluate cytoxicity of Au-NP in human intestinal cells as well as its toxicity after repeated-dose oral administration to rats. Moreover, biokinetics, such as plasma concentration-time profile and biodistribution, was assessed in rats following oral administration. Finally, biological fate of Au-NP was also determined by performing comparative toxicity and biokinetic studies with Au ions.

## 2. Results and Discussion

### 2.1. Characterization of Au-NP

**Figure 1:**
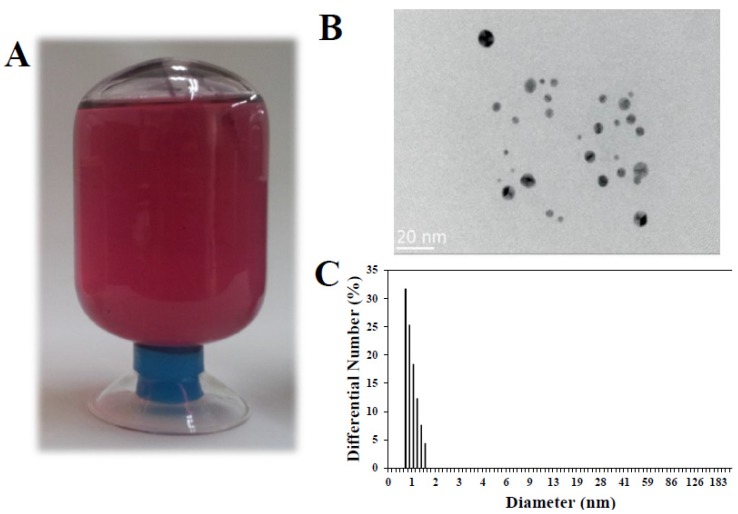
(**A**) Photographic images of Au-NP prepared in 20 mM sodium citrate, 10 mM citric acid monohydrate, and 0.01% poly (vinylpyrrolidone) (PVP) in deionized water (DI); (**B**) Scanning transmission electron microscopic (STEM) image of Au-NP; (**C**) Size distribution of Au-NP measured with zeta potentiometer.

Colloidal Au-NPs produced by an electrolysis were purchased and characterized. Figure 1 showed that Au-NP had a spherical shape (Figure 1B) and a homogeneous size distribution ranged from 5 to 15 nm (Figure 1C), showing red color resulting from nanoparticulated size (Figure 1A). Zeta potential of Au-NP was determined to be slightly negatively charged, −3.37 ± 0.28 mV. Under this condition, the maximum concentration obtained of Au-NPs was 130 μg/mL, and thus, this concentration was used as a stock solution in all the experiments and diluted in an appropriate medium just before treatment.

### 2.2. Cytotoxicity

**Figure 2:**
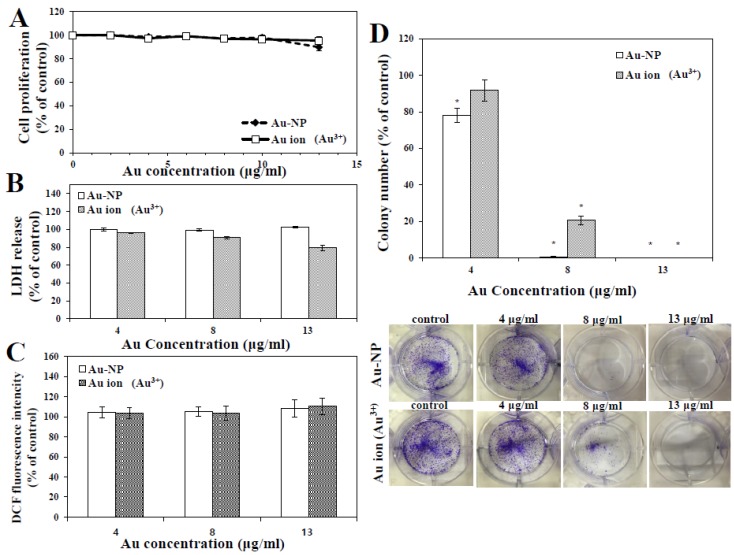
(**A**) Effect of Au-NP or Au ions on cell proliferation of INT-407 cells after 24 h exposure; (**B**) Lactate dehydrogenase (LDH) release from INT-407 cells incubated Au-NP or Au ions for 24 h; (**C**) Reactive oxygen species (ROS) generation in INT-407 cells exposed to Au-NP or Au ions for 24 h; (**D**) Effect of Au-NP or Au ions on colony-forming ability after incubation for seven days. * denote significant difference from untreated controls (*p* < 0.05). Abbreviation: LDH, lactate dehydrogenase; ROS, reactive oxygen species.

Effect of Au-NP on cell proliferation, measured with WST-1 assay, is shown in Figure 2A. To determine whether toxicity of Au-NP is related to its particulate form or oxidized ionic Au form under cell culture condition, equivalent molar amount of Au ions was also used for comparative study in all experiments. Human intestinal cell line (INT-407) was chosen in the present study to correlate cytotoxicity result with *in vivo* oral toxicity. Au-NP or Au ions did not inhibit cell proliferation of INT-407 cells up to 13 μg/mL, the highest concentration tested, after 24 h exposure. Cell proliferation was not affected by Au-NP or Au ions even after 48 h treatment as well (data not shown). Membrane damage caused by nanoparticles was estimated with the lactate dehydrogenase (LDH) release assays. LDH is a cytosolic enzyme in normal cells, which is released into cell culture medium upon cell lysis. As shown in Figure 2B, neither Au-NP nor Au ions significantly cause LDH leakage, which is in good agreement with the result on cell proliferation (Figure 2A). These results indicate that Au-NP has no effects on cell proliferation and cell membrane damage. Commercially available Au-NP was shown to inhibit cell proliferation, as measured with MTT assay, at concentration range from 36 to 1000 ng/mL, depending on cell line tested, demonstrating high toxicity compared to our result [10]. However, physicochemical properties of Au-NP were not presented in the study [10].

Intracellular reactive oxygen species (ROS) induced by nanoparticles was evaluated with carboxy-2',7'-dichlorofluorescein diacetate (H_2_DCFDA). This is a non-fluorescent dye, but becomes green fluorescent in the presence of ROS inside cells. Au-NP or Au ions did not generate intracellular ROS in INT-407 cells up to 13 μg/mL, corresponding to 66 μM (Figure 2C), suggesting that Au-NP did not cause oxidative stress. Colloidal Au-NPs (20 nm, 2 μM) were reported to cause oxidative stress, which is the major mechanism responsible for toxicity in mice [16]. This is not consistent with our result. The controversy between two studies may be related to different synthetic method, particle size, and biological systems tested. It is worth noting that the dispersants used in the present study, citrate and PVP, are currently applied to food additives. Hence, toxicity of Au-NP could be minimized under the synthetic condition. In our experiment, all *in vitro* short-term cytotoxicity results showed that neither Au-NP nor Au ions exhibit cytotoxic effect up to 13 μg/mL. It was report that Au-NPs (18 nm), with various surface modifications, were not toxic up to 200 mM, while ionic Au exhibited cytotoxicity at 25 mM in human leukemia cells [17]. However, cell line and surface chemistry of Au-NP were different from those used in the present study, which may explain different cytotoxicity.

Effect of nanoparticles on long-term cell proliferation was further investigated by colony-forming assay. Figure 2D showed that colony-forming ability was highly suppressed by both Au-NP and Au ions. In particular, inhibition effect of Au-NP was more remarkable than Au ions. It is probable that particulate forms of Au-NP physically covered the surface of cells, and, consequently, disturbed long-term colony formation. High cytotoxic effect of Au-NP on colony-forming ability, compared to short-term cell proliferation, could be related to experimental conditions, such as prolonged exposure time (seven days) and small number (500) of cells exposed. On the other hand, this result suggests that high dose and long-term exposure of both Au-NP and Au ions may suppress cell proliferation.

### 2.3. Biokinetics and Tissue Distribution

**Figure 3:**
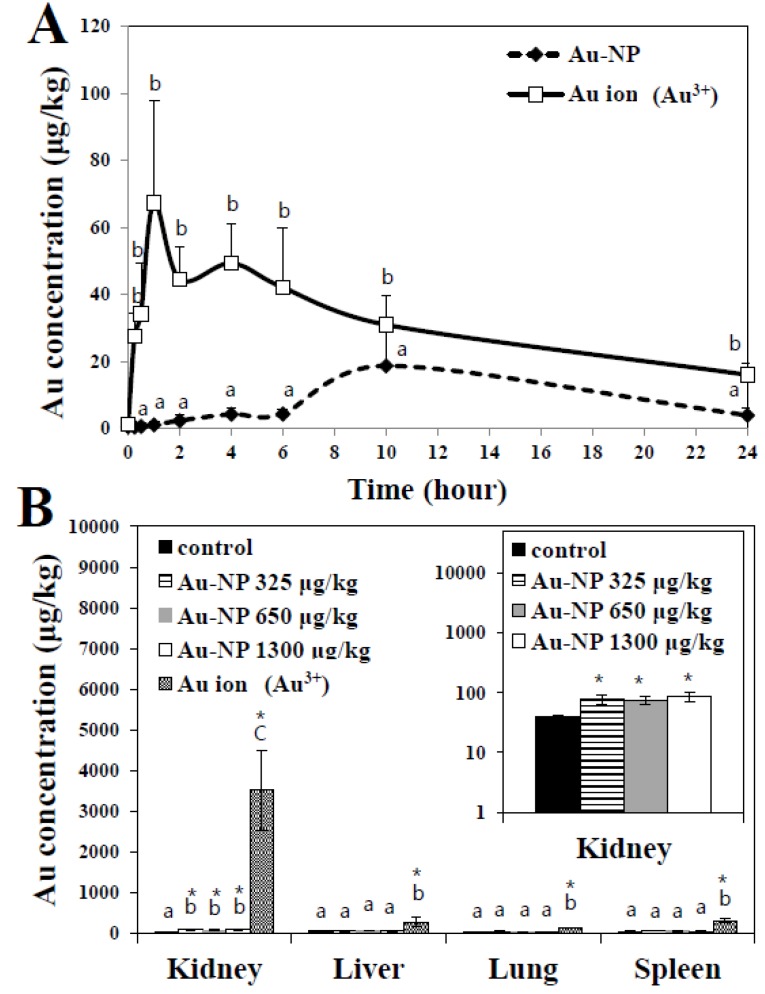
(**A**) Plasma concentration-time profiles of Au-NP or Au ions after a single-dose oral administration to rats; (**B**) Tissue distribution patterns of Au-NP or Au ions after 14-day repeated oral administration to rats. * denote significant difference from untreated controls (*p* < 0.05). Different letters (a, b, c) in figures indicate statistically significant difference at 5%.

Plasma concentration-time profiles of Au-NP or Au ions after a single-dose oral administration to rats are presented in Figure 3A, demonstrating clear difference in biokinetics between two materials. Au-NP slowly entered the blood stream, showing peak concentration at 10 h. Meanwhile, Au ions were rapidly (peak concentration at 1 h) and largely absorbed into the systemic circulation as compared with Au-NP. When biokinetic parameters were calculated (Table 1), significantly higher maximum concentration (*C*_max_) and area under the plasma concentration-time curve (AUC) values for Au ions than those for Au-NP were found. It is worth noting that *C*_max_ and AUC values are important for estimating drug efficacy or toxicity. Oral absorption efficiency, based on AUC values divided by total administered amount, was determined to be 1.85% and 8.54% for Au-NP and Au ions, respectively. This result clearly implies that Au-NP behaves differently from ionic Au at the systemic level, and an extremely small amount of orally administered Au-NP can be absorbed into the body.

**Table 1:** Biokinetic parameters of Au-NP or Au ions after a single-dose oral administration to rats.

Parameters	Au-NP	Au ion (Au^3+^)
*C*_max_ (μg/kg)	18.69 ± 8.33 ^a^	67.35 ± 23.71 ^b^
*T*_max_ (h)	10 ^a^	1 ^b^
AUC (h × μg/kg)	313.12 ± 29.73 ^a^	1443.54 ± 109.23 ^b^
*T*_1/2_ (h)	16.74 ± 1.01 ^a^	15.42 ± 0.51 ^b^
MRT (h)	28.09 ± 2.37 ^a^	30.33 ± 1.65 ^a^
Absorption (%)	1.85 ± 0.18 ^a^	8.54 ± 0.65 ^b^

Abbreviation: AUC, area under the plasma concentration-time curve; MRT, mean residence time; *T*_1/2_, elimination half-life; *C*_max_, maximum concentration; *T*_max_, time to maximum concentration. Absorption (%) was calculated based on AUC values. Different letters (^a^, ^b^) in biokinetic values are indicated when there is significant difference between Au-NP and Au ions (*p* < 0.05).

Tissue distribution study revealed that biodistribution patterns between Au-NP and Au ions were completely different. Au levels significantly increased in kidney, but not in liver, lung, and spleen after 14-day repeated oral administration of Au-NP to rats (Figure 3B), indicating that kidney seems to be a main target organ of orally administered Au-NP. Meanwhile, increased Au levels in kidney suggest that renal excretion can play a role in the elimination of Au-NP from the body. On the other hand, highly elevated Au concentrations were found in kidney, liver, lung, and spleen following oral administration of Au ions, compared to Au-NP-treated groups, indicating different biokinetic behaviors of Au-NP from those of Au ions. It is, therefore, likely that Au-NP remains as particulate form rather than ionic Au form in the body. When only tissue distribution result was considered, it is probable that Au ions have more toxic effects than Au-NP because they highly accumulate in organs and need more time to be eliminated from the organs. It may be associated with high reactivity of Au ions (Au^3+^) compared to Au-NP. A few studies were performed to determine biokinetics and tissue distribution of Au-NPs. Hillyer *et al.* [18] demonstrated that 4 nm colloidal Au-NP were found in kidney, lung, liver, and spleen at high levels in mice following *ad libitum* oral intake of Au-NP in drinking water for seven days, while 10 nm particles accumulated slightly in kidney, showing size-dependent tissue distribution, which seems to be comparable to our result. Fraga *et al.* [19] recently demonstrated that citrate- and pentapeptide-coated Au-NP (20 nm, ~700 μg/kg) were rapidly removed from the blood stream and accumulated mainly in liver after a single-dose intravenous injection to rats. Intraperitoneally injected colloidal Au-NP (12.5 nm, 40–400 μg/kg/day for 8 consecutive days) was reported to be taken up by spleen, kidney, and liver in mice [20]. It is clear that biokinetics of Au-NP differs from exposure route, dose, animal models, and physicochemical properties.

### 2.4. In Vivo Toxicity

**Figure 4:**
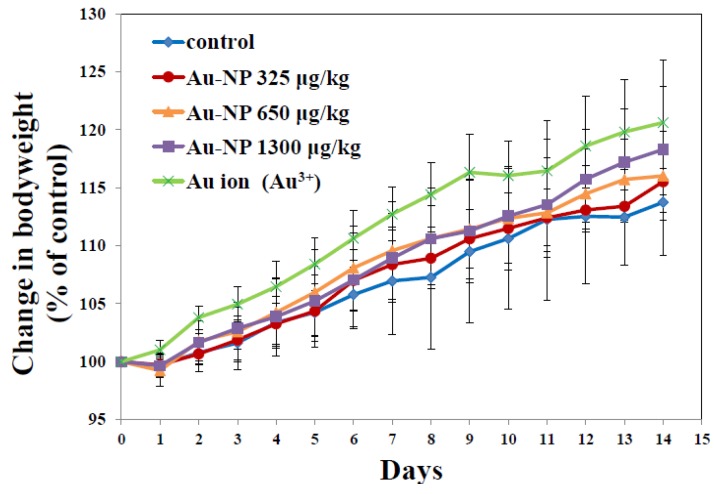
Changes in body weight of rats administered Au-NP or Au ions during 14-day consecutive oral administration.

*In vivo* toxicity of Au-NP was assessed in rats following 14-day repeated dose oral administration. Changes in body weight during the treatment are presented in Figure 4. No significant decrease in body weight was observed in rats administered Au-NP up to 1300 μg/kg. Moreover, Au ions did not affect body weight gain when equivalent molar amount of Au ions was treated. Organo-somatic indices demonstrated that organ weight did not change by the treatment of Au-NP, supporting its low toxicity (Table 2). Significant increase in organo-somatic index was observed only in small intestine administered Au ions.

Hematological analysis data revealed that all hematological values did not significantly change in rats administered Au-NP or Au ions as compared to untreated control (Table 3), indicating their low toxicity. Serum biochemical analysis demonstrated that glucose (GLU) level significantly increased only in the serum of rats treated with medium dose of Au-NP (Table 4). However, dose-dependent decrease or increase in GLU levels was not found.

**Table 2:** Organo-somatic indices of rats after 14-day repeated oral administration.

Organ	Au-NP				Au ion (Au^3+^)
	Control	1300 μg/kg	650 μg/kg	325 μg/kg	1300 μg/kg
Brain	0.72 ± 0.07	0.72 ± 0.06	0.81 ± 0.05	0.83 ± 0.09	0.76 ± 0.04
Kidney	0.90 ± 0.09	0.89 ± 0.06	0.90 ± 0.03	0.88 ± 0.10	0.88 ± 0.05
Large intestine	1.63 ± 0.25	1.46 ± 0.21	1.32 ± 0.23	1.62 ± 0.25	1.58 ± 0.37
Liver	4.46 ± 0.59	4.51 ± 0.31	4.93 ± 0.43	4.59 ± 0.39	4.52 ± 0.60
Lung	0.49 ± 0.04	0.51 ± 0.07	0.60 ± 0.15	0.47 ± 0.03	0.53 ± 0.09
Ovary	0.05 ± 0.01	0.05 ± 0.01	0.05 ± 0.003	0.04 ± 0.01	0.05 ± 0.01
Small intestine	4.04 ± 0.29	3.90 ± 0.25	3.46 ± 0.33	3.50 ± 0.35	3.82 ± 0.24 *
Spleen	0.25 ± 0.02	0.29 ± 0.02	0.27 ± 0.02	0.25 ± 0.05	0.28 ± 0.02
Stomach	2.44 ± 0.90	3.17 ± 0.71	2.36 ± 0.60	1.76 ± 0.52	2.51 ± 0.63

* Denote significant difference from untreated controls (*p* < 0.05).

**Table 3:** Hematological values after 14-day repeated oral administration of Au-NP or Au ions to rats.

Dose		WBC	WBC Differential Counting (%)					RBC	Hb	HCT	MCV	MCH	MCHC	RETI	PLT	PT	APTT
		(10^3^/μL)	NE	LY	MO	EO	BA	(10^6^/μL)	(g/dL)	(%)	(fL)	(pg)	(g/dL)	(%)	(10^3^/μL)	(s)	(s)
**Control**		8.7	7.8	87.4	1.8	1.4	0.6	7.8	15.1	49.3	63.0	19.2	30.5	2.1	785	14.3	32.3
		±4.4	±2.5	±1.9	±0.2	±0.2	±0.2	±0.2	±0.4	±1.1	±1.1	±0.3	±0.2	±0.3	±457	±0.4	±8.3
**Au-NP**	**1300 μg/kg**	11.3	11	84.9	1.6	0.8	0.7	7.4	14.4	47.1	63.7	19.5	30.6	2.7	1060	13.9	38.8
		±2.5	±2.4	±2.0	±0.1	±0.2	±0.2	±0.2	±0.3	±1.1	±0.2	±0.1	±0.3	±0.3	±13	±0.1	±5.0
	**650 μg/kg**	9.5	9.8	85.6	1.6	1.2	0.8	7.7	14.8	19.4	64.5	19.3	29.9	2.4	968	18.7	85.9
		±0.3	±1.4	±2.5	±0.3	±0.5	±0.5	±0.1	±0.1	±0.8	±0.6	±0.4	±0.4	±0.9	±151	-	-
	**325 μg/kg**	10.8	7.1	86.1	2.5	1.5	0.7	7.5	14.5	47.4	63.5	19.5	30.6	2.7	1114	13.7	38.8
		±0.4	±1.9	±1.7	±1.1	±0.2	±0.3	±0.3	±0.3	±1.4	±0.3	±0.3	±0.3	±0.7	±177	±0.5	±1.6
**Au Ion (Au^3+^)**	**1300 μg/kg**	10.2	9.6	85.5	2.0	1.3	0.7	8.1	15.5	51.9	64.5	19.2	29.8	2.8	1068	14.4	43.5
		±1.4	±1.6	±1.8	±0.7	±0.3	±0.2	±1.0	±1.1	±4.7	±2.3	±1.0	±0.6	±0.7	±57	±0.3	±2.1

Abbreviation: WBC, total leucocyte count; NE, neutrophils; LY, lymphocytes; MO, monocytes; EO, eosinophils; BA, basophils; RBC, total erythrocyte count; Hb, hemoglobin concentration; HCT, hematocrit; MCV, mean cell volume; MCH, mean cell hemoglobin; MCHC, mean cell hemoglobin concentration; RETI, reticulocyte; PLT, platelet; PT, prothrombin time; APTT, activated partial thromboplastin time. All treated groups showed no significant differences from the untreated control group (*p* > 0.05).

**Table 4:** Serum biochemical values after 14-day repeated oral administration of Au-NP or Au ions to rats.

**Dose**		**TP**	**ALB**	**A/G**	**T-BIL**	**ALP**	**AST**	**ALT**	**CREA**	**BUN**
		**(g/dL)**	**(g/DL)**	**-**	**(mg/dL)**	**(U/L)**	**(U/L)**	**(U/L)**	**(g/dL)**	**(mg/dL)**
**Control**		7.5	4.8	1.5	0.1	906	99	60	0.4	25.0
		±0.2	±0.1	±0.1	±0.0	±54	±13	±13	±0.1	±4.3
**Au-NP**	**1300 μg/kg**	7.5	4.6	1.6	0.1	878	99	52	0.4	23.3
		±0.3	±0.1	±0.1	0.0	±236	±5	±6	±0.0	±3.1
	**650 μg/kg**	7.6	4.7	1.6	0.1	812	84	45	0.5	25.1
		±0.5	±0.2	±0.2	±0.0	±85	±9	±9	±0.2	±0.4
	**325 μg/kg**	7.2	4.6	1.7	0.1	908	89	46	0.6	24.0
		±0.1	±0.1	±0.1	±0.0	±102	±4	±8	±0.0	±1.5
**Au Ion (Au^3+^)**	**1300 μg/kg**	7.2	4.6	4.6	0.1	766	82	48	0.6	25.2
		±0.2	±0.1	±0.1	±0.0	±159	±4	±7	±0.1	±3.0
**Dose**		**CHOL**	**TG**	**GLU**	**CA**	**IP**	**CK**	**Na**	**K**	Cl
		**(mg/dL)**	**(mg/dL)**	**(mg/dL)**	**(mg/dL)**	**(mg/dL)**	**(lU/L)**	**(mmoL/L)**	**(mmoL/L)**	**(mmoL/L)**
**Control**		109	119	568	14.8	9.7	347	144	9.3	96
		±5	±3	±21	±0.2	±0.3	±135	±2	±1.1	±1
**Au-NP**	**1300 μg/kg**	107	131	644	15.2	10.8	255	146	8.8	95
		±16	±11	±38	±0.5	±0.6	±92	±2	±0.7	±2
	**650 μg/kg**	113	117	718 *	15.2	11.0	122	144	9.5	95
		±26	±32	±23	±0.5	±0.8	±38	±2	±0.6	±1
	**325 μg/kg**	98	99	524	14.3	8.8	142	147	7.0	97
		±3	±29	±34	±0.4	±1.0	±65	±2	±0.6	±2
**Au Ion (Au^3+^)**	**1300 μg/kg**	105	86	549	14.0	9.8	119	146	7.4	97
		±4	±13	±95	±0.7	±0.3	±24	±2	±1.1	±1

Abbreviation: TP, total protein; ALB, albumin; A/G, A/G ratio; T-BIL, total bilirubin; ALP, alkaline phosphatase; AST, aspartate aminotransferase; ALT, alanine aminotransferase; CREA, creatinine; BUN, blood urea nitrogen; CHOL, total cholesterol; TG, triglycerides; GLU, glucose; CA, calcium; IP, inorganic phosphorus; CK, creatine kinase; Na, sodium; K, potassium; Cl, chloride. * denote significant difference from untreated controls (*p* < 0.05).

**Figure 5:**
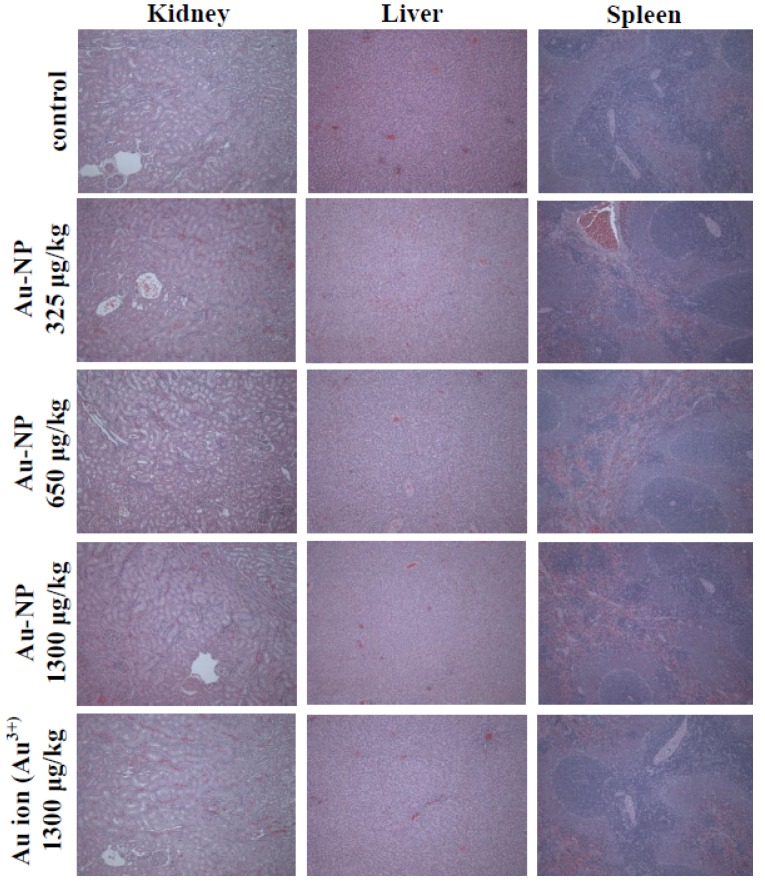
Histopathologial examination of organs after 14-day repeated oral administration of Au-NP or Au ions to rats.

Toxicological effects of Au-NP in rats were further confirmed by histopathological examination. Figure 5 shows that no abnormal or histopathological findings were observed in liver, kidney, and spleen after 14-day repeated administration of Au-NP or Au ions. This result clearly showed that Au-NP or Au ions did not cause toxic effects. Although a decreased serum biochemical value was found in 650 μg/kg Au-NP-treated group in terms of glucose metabolism, pathological lesion related to the test materials was not observed in any organs treated. Furthermore, dose-dependent toxicity of Au-NP was not found. Hence, all the *in vivo* toxicity results suggest that Au-NP did not cause toxicological effects up to 1300 μg/kg, following 14-day consecutive oral administration to rats. It is worth noting that expected exposure dose of Au-NP in biomedical application is far less than 1300 μg/kg, the highest dose used in this study [21]. Pentapeptide-coated Au-NP was reported to have more toxicity than citrate-coated Au-NP (20 nm, ~700 μg/kg), showing mild anemia and spleen atrophy on 28 days post-single intravenous injection [19]. Au-NPs in the size range from 8 to 37 nm exhibited toxicity after intraperitoneal injection to mice at a dose of 8 mg/kg/week, demonstrating pathological abnormalities in liver, lung, and spleen [22]. These *in vivo* toxicity results are not comparable to our results, because experimental conditions such as exposure route, dose treated, and physicochemical properties of Au-NP were different. Taken together, Au-NP used in the present study did not cause severe oral toxicity in rats.

## 3. Experimental Section

### 3.1. Materials and Characterization

Colloidal Au-NP was purchased from SMNANOBIO Co., Ltd. (Daejeon, Korea), which was prepared by an electrolysis method, as described previously [23]. Briefly, two pure gold plates (5 cm × 10 cm × 2 cm) were used as electrodes. The two electrodes were placed vertically fact-to-face at a constant distance of 20 mm. Electrolyte consisted of 20 mM sodium citrate, 10 mM citric acid monohydrate, and 0.01% PVP (Mw = 29,000 g/M, Sigma-Aldrich, St. Louis, MO, USA) in DI. A pH range of 6.5–7.5, electrolyte temperature of 80–90°C and current of 20–30 Å were applied to obtain the Au-NPs. After 30 min, well-dispersed colloidal Au-NPs were fabricated in DI. The nanoparticles were then analyzed by STEM (JEM-2100F, JEOL, Co., Tokyo, Japan) and zeta potentiometer (ZEN3600, Malvern Instruments Ltd., Worcestershire, UK), respectively.

### 3.2. Cell Culture

Human intestinal epithelial cells (INT-407) were provided by Dr. Tae-Sung Kim at Korea University (South Korea) and cultured MEM (Welgene, Gyeongsangbuk-do, Korea) under a humidified atmosphere (5% CO_2_/95% air) at 37°C. The medium was supplemented with 10% heat inactivated fetal bovine serum, 100 units/mL penicillin, and 100 μg/mL streptomycin (Welgene).

### 3.3. Cell Proliferation

The effects of colloidal Au-NP on cell proliferation were measured with the WST-1 (Roche, Molecular Biochemicals, Mannheim, Germany). Briefly, cells (5 × 10^3^ cells/100 μL) were exposed to 2 to 13 μg/mL Au-NP or equivalent molar amount of HAuCl_4_ (Au^3+^) (Sigma-Aldrich) based on Au content for 24 h. Then, 10 μL of WST-1 solution (Roche) was added to each well, and cells were incubated for a further 4 h. Absorbance was then measured using a plate reader at 440 nm (SpectraMax^®^ M3, Molecular Devices, Sunnyvale, CA, USA). Cells incubated in the absence of Au-NP were used as controls.

### 3.4. LDH Leakage Assay

The release of LDH was monitored with the CytoTox 96 Non-Radioactive Cytotoxicity assay (Promega, Madison, WI, USA). Cells (4 × 10^4^ cells/1 mL) were incubated with 4 to 13 μg/mL Au-NP or equivalent molar amount of Au ions for 24 h. Then, the plates were centrifuged, and aliquots (50 μL) of cell culture medium were collected from each well and placed in new microtiter plates. Then, 50 μL of substrate solution was added to each well and the plates were further incubated for 30 min at room temperature. Finally, after adding the 50 μL of stop solution, the absorbance at 490 nm was measured with a microplate reader (SpectraMax^®^ M3, Molecular Devices, Sunnyvale, CA, USA). Cytotoxicity is expressed relative to the basal LDH release from untreated control cells.

### 3.5. Intracellar ROS Production

Intracellular ROS levels were monitored using a peroxide-sensitive fluorescent probe, carboxy-2',7'-dichlorofluorescein diacetate (H_2_DCFDA) (Molecular Probes Inc., Eugene, OR, USA), according to the manufacturer’s guidelines. Briefly, cells (5 × 10^3^ cells/100 μL) were incubated with Au-NP or equivalent molar amount of Au ions for 24 h under standard conditions as described above, washed with phosphate buffered saline (PBS), and incubated with 40 μM carboxy-H_2_DCFDA for 60 min at 37°C. After washing with PBS, DCF florescence was immediately measured using a fluorescence microplate reader (SpectraMax^®^ M3, Molecular Devices) and excitation and emission wavelengths of 490 nm and 535 nm, respectively. Cells not treated with nanoparticles were used as controls.

### 3.6. Clonogenic Assay

Cells (5 × 10^2^ cells/2 mL) were seeded in 6-well plates and incubated overnight at 37 °C under a 5% CO_2_ atmosphere. The medium in the plates was then replaced with fresh medium containing various concentration of Au-NP (4, 8, or 13 μg/mL) or equivalent molar amount of Au ions and incubation continued for 7 days. For colonies counting, cells were washed with PBS, fixed with 90% crystal violet solution (Sigma-Aldrich) for 1 h. After cells were washed with deionized water and air-dried, colonies consisted of more than 50 cells were counted. Each experiment was done triplicate and colony number in the absence of Au-NP was used as a control.

### 3.7. Animals

Five-week-old female Sprague Dawley (SD) rats weighing 90–100 g were purchased from Nara Biotech Co., Ltd. (Seoul, Korea). Animals were housed in plastic animal cages in a ventilated room maintained at 20 ± 2 °C and 60 ± 10% relative humidity under a 12 h light/dark cycle. Water and commercial laboratory complete food for rats were made available *ad libitum*. Animals were acclimated to this environment for 7 days before treatment. All animal experiments were performed after obtaining approval from the Animal and Ethics Review Committee of Seoul Women’s University.

### 3.8. Biokinetics

Six female rats per group were administered a single dose of 1300 μg/kg Au-NP or equivalent molar amount of Au ions by oral gavage; controls (*n* = 5) received an equivalent volume of dispersed solution containing sodium citrate, citric acid, and PVP as described in Materials and Characterization Section. To determine plasma Au concentrations, blood samples were collected via a tail vein at 0, 0.25, 0.5, 1, 2, 4, 6, 10, and 24 h. Blood samples were centrifuged at 3000× *g* for 15 min at 4°C to obtain plasma. The following biokinetic parameters were estimated using Kinetica version 4.4 (Thermo Fisher Scientific, Waltham, MA, USA): Maximum concentration (*C*_max_), time to maximum concentration (*T*_max_), area under the plasma concentration-time curve (AUC), half-life (*T*_1/2_), and mean residence time (MRT).

### 3.9. Oral Toxicity Assessment and Tissue Distribution

Prior to dosing, food, but not water, was withheld for 4 h. Five female rats per group were administered of 1300 μg/kg of Au-NP or equivalent molar amount of Au ions daily for 14 consecutive days by oral gavage; controls (*n* = 5) received an equivalent volume of dispersed solution containing sodium citrate, citric acid, and PVP. Body weight changes, behaviors, and symptoms were carefully recorded daily after administration. At the end of experiment, animals were sacrificed by CO2 euthanasia, organs were collected, and organ weights of all treated rats were measured. Organo-somatic index was calculated by the following formula:

[Weight of the organ (g)/Total body weight (g)] × 100

Blood samples were collected from the posterior vena cava for hematology and serum biochemistry analysis. Whole blood samples was drawn into an EDTA 3K tube (BD Biosciences, Franklin Lakes, NJ, USA) and applied to an automatic analyzer (ADVIA120E, Bayer, New York, NY, USA) for hematology. For coagulation analysis, whole blood samples were transferred into a vacutainer (sodium citrate 3.2%, BD Bioscience), centrifuged at 3000 rpm for 10 min, and the plasma was used to determine aggregation time using coagulometer (ALC 7000, Werfen Medical, IL, USA). For serum biochemical analysis, serum samples were applied to an automatic biochemical analyzer (TBA-120FR, Otawara, Toshiba, Japan). Histopathological examination was performed on liver, kidney, and spleen fixed with 10% neutral buffer formalin and stained with hematoxylin and eosin.

For the tissue distribution study, another female rats (*n* = 5) were orally administered daily for 14 consecutive days, and samples of kidney, liver, lung, and spleen were collected on the 14th day.

### 3.10. ICP-MS Analysis

Biological samples (0.2 g of plasma and total tissues) were pre-digested in 15–20 mL of ultrapure nitric acid overnight and heated at ~160 °C. Aqua regia solution (HCl:HNO_3_ = 3:1, 15–20 mL) was then added in order to change metallic gold to gold chloride ions, and mixtures were heated until samples had been completely digested. When the remaining solution is 3 mL, the solution was transferred to another vial, and double distilled water was added to a total mass of 10 g. All biological samples were spiked with known concentrations of standard Au solution, which were also used for the external calibration. Analyses of total Au contents were conducted by ICP-MS (ELAN 6100, Perkin-Elmer SCIEX, Norwalk, CT, USA).

### 3.11. Statistical Analysis

Statistical analysis was performed using the Student’s t test for unpaired data and one-way analysis of variance (ANOVA) was conducted using SAS software (Tukey’s Test, Version. 11.0, SAS Institute Inc., Cary, NC, USA) to determine the significances of differences between experimental groups. All results are presented as means ± standard deviations and *p* values of less than 0.05 were considered significant.

## 4. Conclusions

Toxicity of colloidal Au-NP was evaluated in human intestinal cells and *in vivo* rats after 14-day repeated oral administration. Toxicity of Au-NP was also compared to Au ions in all experiments. The results demonstrated that Au-NP or Au ions did not cause cytotoxic effects on INT-407 cells after 24 h exposure in terms of inhibition of cell proliferation, membrane damage, and ROS generation. However, when a small number of cells were exposed to Au-NP or Au ions for more prolonged time, seven days, colony forming ability remarkably decreased by both Au-NP and Au ion treatments, suggesting their potential toxicity after long-term exposure at high concentration. Plasma concentration-time profiles showed that extremely small amount of orally administered Au-NP was slowly absorbed into the blood stream, while relatively high and rapid oral absorption of Au ions was found. Tissue distribution study revealed that Au-NP slightly accumulated only in kidney, whereas high Au levels were detected in kidney, liver, lung, and spleen after oral administration of Au ions. These biokinetic results suggest that biological fate of Au-NP is primarily in nanoparticulate form, not in ionic Au form, at the systemic levels. *In vivo* experiment showed that Au-NP did not cause toxicological effects on rats following 14-day consecutive oral administration. Further toxicity study of Au-NP after long-term exposure is necessary to confirm its low toxicity.
